# Understanding the dynamics of sustainable change: A 20-year case study of integrated health and social care

**DOI:** 10.1186/s12913-018-3061-6

**Published:** 2018-06-04

**Authors:** Charlotte Klinga, Henna Hasson, Magna Andreen Sachs, Johan Hansson

**Affiliations:** Department of Learning, Informatics, Management and Ethics (LIME), Medical Management Centre (MMC) | Karolinska Institutet, 171 77 Stockholm, Sweden

**Keywords:** Implementation, Organisational sustainability, Change management, Integrated care, Mental health

## Abstract

**Background:**

Change initiatives face many challenges, and only a few lead to long-term sustainability. One area in which the challenge of achieving long-term sustainability is particularly noticeable is integrated health and social care. Service integration is crucial for a wide range of patients including people with complex mental health and social care needs. However, previous research has focused on the initiation, resistance and implementation of change, while longitudinal studies remain sparse. The objective of this study was therefore to gain insight into the dynamics of sustainable changes in integrated health and social care through an analysis of local actions that were triggered by a national policy.

**Methods:**

A retrospective and qualitative case-study research design was used, and data from the model organisation’s steering-committee minutes covering 1995-2015 were gathered and analysed. The analysis generated a narrative case description, which was mirrored to the key elements of the Dynamic Sustainability Framework (DSF).

**Results:**

The development of inter-sectoral cooperation was characterized by a participatory approach in which a shared structure was created to support cooperation and on-going quality improvement and learning based on the needs of the service user. A key management principle was cooperation, not only on all organisational levels, but also with service users, stakeholder associations and other partner organisations. It was shown that all these parts were interrelated and collectively contributed to the creation of a structure and a culture which supported the development of a dynamic sustainable health and social care.

**Conclusion:**

This study provides valuable insights into the dynamics of organizational sustainability and understanding of key managerial actions taken to establish, develop and support integration of health and social care for people with complex mental health needs. The service user involvement and regular reviews of service users’ needs were essential in order to tailor services to the needs. Another major finding was the importance of continuously adapting the content of the change to suit its context. Hence, continuous refinement of the change content was found to be more important than designing the change at the pre-implementation stage.

## Background

Because organisational change requires investments of time, money and human resources, the lasting impact of this type of change is generally of great interest to care providers, funders and other stakeholders. Change initiatives, however, face many challenges, and only a few of them lead to long-term sustainability [1]. Knowledge of whether program outcomes are beneficial and sustainable is also valuable when spreading and supporting such programs across several settings [2]. In the health sector, the prior research has mainly been focused on the early stages of the change process – initiation, resistance and implementation [3–5] – and longitudinal studies are sparse [1, 5, 6]*.* The research on strategies for achieving sustainability in change initiatives is rather scant [7], and studies that explicitly address sustainability are almost absent [8]. Therefore, knowledge on how to achieve long-term maintenance of organisational changes is needed [2, 9].

The recent research highlights several factors’ influences on sustainability, including the content of the change and its contextual, political, organisational, processual and temporal factors [5, 10]. Certain enabling factors have been highlighted, including a supportive context, capacity building, effective relationships among the actors, and rigorous planning and decision making [11]. However, few investigations of these factors or of the possible interrelationships between them have been conducted [12]. Thus, the ways in which these factors interact and impact sustainable change remain unclear.

A conventional way to study sustainability is to describe the extent to which the change content is maintained over a period of time – for instance, after initial or external support is removed [2, 13]. This approach assumes that the change content is constant over time. However, adaptations to changes in local conditions are common and sometimes necessary. Thus, interventions are rarely implemented as they were originally intended, and their content often varies over time [14]. This makes the concepts of fidelity and adaptation to intervention important in sustainability studies [13]. These two concepts are inherently linked [15] ways of overcoming organisational inertia while adapting to contemporary environmental changes [16].

Chambers [13] recently proposed a more dynamic view on sustainability called the Dynamic Sustainability Framework (DSF); in this view, change is an ongoing adaptation process in which the intervention is continuously refined and improved in relation to its context. The context comprises the practice setting, its surroundings and the ecological system [13]. Thus, the DSF addresses the paradox of sustainability amid ongoing change. The fit between the intervention and the local context can be optimized by continuously matching the characteristics of the intervention to the practice setting and the ecological system through appropriate improvements [13]. This is an iterative, dynamic process in which continued learning and development are central. Thus, ongoing quality improvement is the ultimate goal of such interventions.

One area in which the challenge of achieving long-term sustainability is particularly noticeable is integrated health and social care. In the contemporary system, which was constructed to provide diverse, unconnected health and social care services, those who protect these systems’ separate regulations and policies may constantly challenge the idea that the systems should be integrated [17]. Additional challenges include organisations’ conflicting objectives and values as well as professionals’ distinct cultures [18]. Hence, there is neither a universal definition of integrated care nor a one-size-fits-all model [19, 20]. However, evidence has demonstrated that integrated health and social care leads to care that is more people-centric and holistic; this can be achieved by developing cross-sectoral and inter-professional collaboration [21]. Such services is of great value for people who have complex health and social care needs, such as those who are suffering from mental illness. One major objective that the World Health Organisation (WHO) set forth in its *Mental Health Action Plan 2013-2020* [22] was to provide *comprehensive, integrated mental health and social care services in community-based settings*. Hence, innovative approaches are needed in both technical and relational aspects [23]. To facilitate long-term organisational sustainability, policies must address the entire health system, including incentive structures and performance measures [17]. A 2016 review concluded that no complete, peer-evaluated longitudinal studies have been conducted on integrated health and social care [24], so the uncertainty regarding long-term effects remains.

In Sweden, the responsibility for the provision of health and social care services is managed on three levels: the government (national level), the 21 county councils (regional level) and the 290 municipalities (local level). The county councils have the responsibility for provision of health care and the municipalities for the provision of care for elderly and disabled people, as well people in need of long-term mental health care. Municipalities and county councils have a substantial freedom to organise health and social care services [25]. Since the 1990s several structural changes have aimed to move from inpatient care towards outpatient care [26]. That was also the case in 1995 when Sweden launched a national policy which was manifested in the Health and Medical Care Act, Social Services Act and The Swedish Act concerning Support and Service for Persons with Certain Functional Impairments to create more integrated services for people with mental illnesses. The policy’s goals were to improve the coordination and individualisation of care and by service user involvement integrate them into society. The policy clarified the municipality’s responsibility for planning and coordinating interventions and for developing housing and employment for those who suffer from mental disorders. As before, the county council’s responsibilities were the prevention, investigation and treatment of psychiatric conditions [27]. The continued sectoral division of care and support meant that the county and municipal social care organisations have to develop cooperation to meet the service users’ complex needs. However, the authorities in Sweden interpreted the policy differently.

More than 20 years have passed since the policy was introduced. In that time, much has changed, but the division of responsibilities between the municipality and the county remains a key challenge in providing cohesive health and social care [28]. A recent study found that, at a policy level, establishing an overall perspective on healthcare and social support for people with mental illness has been difficult. The reasons for this include shortcomings in cooperation between the levels of care and inadequate coordination between social services, primary care, employment services and the nation’s social insurance agency. The focus remains split among various components rather than on the whole picture [29].

Nevertheless, examples of sector-related barriers being overcome exist. For instance, one geographical area has sustained its extensive integration of mental health and social care services since the policy was implemented [30]. For the purpose of this paper we use the definition from WHO for integrated health services [18].

“Integrated health services are health services that are managed and delivered in a way that ensures people receive a continuum of health promotion, disease prevention, diagnosis, treatment, disease management, rehabilitation and palliative care services, at the different levels and sites of care within the health system, and according to their needs throughout their life course ”.

The abbreviation IC will be used, referring to integrated mental health and social care services. Based on our previous research [30–32], we anticipated that this case would be particularly interesting to study from a sustainability perspective. By viewing sustainability as a process in a constantly changing context, the DSF is a relevant means through which to explore the mechanisms of sustainability in this integrated mental health and social care organisation.

The specific objective of this study is to gain insight into the dynamics of sustainable changes in integrated health and social care through an analysis of local actions that were trigged by a national policy.

## Methods

### Design

To study inter-sectoral cooperation, a retrospective and qualitative case-study research design was applied based on data from the model organisation’s steering-committee minutes from 1995 through 2015.

### Study setting

The area of Sweden in which the inter-sectoral cooperation took place has a long record of providing integrated health and social care, with the county council providing psychiatric care and the municipality providing social services. The population in this urban area is just over 96,000; the proportion of the population with a foreign background is 51%; the average age is 39 years; the employment rate of 20-64 years is 72% [33]. This case was chosen because it represented a unique example of long-lasting, inter-sectoral cooperation. Since the introduction of the national psychiatric policy, the cooperating agencies have striven to overcome fragmented health and social care. Through the policy, the obligation of people for those with long-term psychiatric disorders upon their discharge from closed-environment mental health institutions was transferred from the county council to the municipality. During the policy’s establishment period (1996-1998), funding was available for both the county council and the municipality, provided that they presented a shared plan for how the money would be used to facilitate organisational and operational changes.

The original idea behind this integration was to create a single point at which all service users could receive help and support, regardless of which authority had the competence or means to address the problem. Initially, this plan was to offer services to those over 18 years old who had chronic, severe mental illnesses that caused permanent disabilities, as well as to those who were in need of both psychiatric treatment and social services. Today, the integration has evolved to also include people with neuropsychiatric diagnoses, people with addiction problems and other target groups. Since 1995, a steering committee has governed the integrated organisation, with representatives from the county council’s psychiatric care and the municipality’s social services. The committee’s mission is to *develop cooperation between the municipality’s social care and the county council’ psychiatric care*. The services consist of geographically dispersed co-located centres and mobile units for specific target groups. Although separate legislation regulates the services, they are all organised based on inter-professional teams and are managed, at all levels, through co-leadership of two leaders, one from each organisation. Access to services is mainly via primary care but also from emergency care. Inpatient care is provided by the regional hospital.

### Data collection and data characteristics

Data were collected from the minutes of all steering-committee meetings held from 1995 through 2015. The minutes were available in digital form (*n* = 98; approximately 3-5 meetings per semester, fewer in recent years) and followed the same basic structure. However, some variation regarding the content occurred due to the relevance of certain topics and questions at certain times. This variation in content determined the committee’s composition; in addition to the permanent members – representatives from the county, the municipality and the stakeholder associations (e.g. patients’ and relatives’ associations) – other relevant stakeholders were invited. The same person wrote all the minutes, which were primarily for the internal use of the organisation’s managers and professionals. However, the minutes were also available to cooperating partners outside the organisation and to other interested stakeholders.

### Data analysis

Thematic analysis, as Braun and Clarke [34] described, is used to handle extensive data sets. This study’s data included a large amount of information generated over twenty years, which required condensation; a semantic approach was used to handle this rich data set. Based on the study’s objective, an initial orientation was developed, and a search for codes and meaningful groups was performed. Based on the predetermined area of interest, actions not directly linked to integration were excluded. This first stage of the analysis was explorative and inductive [35]; data were used to map the steering committee’s actions. The resulting analysis was intended to illustrate the trend in integration over time and to serve as a basis for the subsequent analysis in stage 2, which was intended to mirror the findings regarding the key elements of the DSF. This procedure resulted in a detailed data analysis that would aid in identifying the empirical themes from the narrative description. The thematic analysis-process is illustrated in Table 1.

**Table 1:** Stages of the thematic analysis

Stage 1	Description of the process
1. Learn about the data set 2. Create an initial code list 3. Group the codes 4. Search for themes 5. Create a timeline	Read and reread data, take notes and mark ideas for coding Organise data into meaningful codes based on potential interest Search for relationships between codes to create sub-themes Sort codes and sub-themes into data-dependent themes Extract activities related to the area of interest using a timeline
Stage 2	Description of the process
6. Investigate using theory 7. Search for themes 8. Refine the themes	Analyse the data through specific questions based on DSF Sort codes into theory-dependent themes related to integration Consider the coded data extracts and the individual themes in relation to the entire data set

## Results

The results are presented in two parts. The first, *Case findings*, provides a chronological case narrative of the key events related to the integration. The second, *Empirical themes*, is based on identified chains of actions that are reflected in DSF.

### Case findings

The individual organisations had a history of collaboration prior to the launch of the psychiatric policy. That policy, however, triggered the development of an integrated health and social care organisation, with project funding provided for both the county council and the municipality. In 1995, both the inter-sectoral steering committee and the two project managers (one from each organisation) were put in place. One guiding principle was that all service users should be at the centre of – and participate in – the planning of care and support. The integrated organisation started by inventorying the need for staff training and for a cooperative model to apply with each service user. Stakeholder associations were involved, and the central role of the service users (i.e. the patients and clients), their families and the stakeholder associations was emphasised. A co-leadership model was created in which the leaders from each sector jointly managed all services. Joint trainings, information sessions, outdoor excursions and conferences were arranged. The continuous exchange of competencies and experiences was given high priority, and co-run projects targeted to specific groups, such as people with substance abuse, were initiated.

In 1996 and 1997, several changes were introduced to integrate the services. For instance, three co-located and jointly managed centres were opened, mainly for persons with psychosis and complex needs. Furthermore, each service user received coordinators from each organisation; these coordinators shared the responsibility for all of the user’s health and social care planning. Data on the service users’ needs (the Camberwell Assessment of Needs [CAN] scale) were used for these purposes. Individualised care and rehabilitation plans were introduced to personalise care and to support service users. In addition, shared clinical guidelines and agreements concerning financial issues were established. The overarching vision, in which service users would be active co-producers, triggered the development of a consistent terminology; the term ‘customer’, instead of ‘patient’ or ‘client’, was suggested. Nevertheless, the term ‘customer’ was incompatible with then-current national regulations, and the terms ‘patient’ (for psychiatric care) and ‘client’ (for social care) were kept. The importance of a shared IT system was recognised, and much effort was spent in trying to achieve this. However, the Swedish Data Protection Authority did not permit the use of such a shared register for service-users assessment data (using the CAN scale) because of questions regarding data ownership. Thereafter, the possibility of digitally sharing care and rehabilitation plans was examined.

In 1998, challenges related to financing surfaced, partly because the initial funding had ended. In addition, difficulties concerning the management of the integrated services were noted, as differences in managers’ decision-making mandates varied between the organisations. To meet these challenges, additional training in integration was offered; for instance, the two project managers received support in how to manage integrated projects, and the coordinators were trained in case management. The expansion of integration to include new groups of service users (e.g. those with severe mental disabilities) was considered. In addition, a new organisation was proposed that would be aimed at outsourcing cooperative units to the municipal districts, but the steering committee rejected this proposal due to the risk of losing coordination and the knowledge required for rehabilitation in the event of a disruption in the organisation’s integration.

In 1999, the steering committee reviewed the integrated organisation’s costs. Savings from the previous year enabled the financing of additional training to facilitate the handover of service users from external locations to the local municipality’s care. Actions were planned to simplify the practical work and to increase collaboration. For instance, the plan was for decisions about housing support to be transferred to the unit level (i.e. as close to the service users as possible) with the goal of improving collaboration among social workers, assistance officers and occupational therapists. The integrated organisation continued to grow; it initiated a mobile team and developed the coordinator role to enhance focus on the entire rehabilitation process. The shared use of the CAN data was further developed to tailor both health and social care to meet the service users’ individual needs.

In 2000 and 2001, efforts were made to make the two organisations’ economic steering mechanisms equivalent. The steering committee also expanded to include members who represented areas that had recently been integrated, such as elder care. It was agreed that all new projects within the integrated organisation would be managed in close collaboration and based on formal agreements – in contrast to earlier, verbal agreements. An example of concrete integration was the announcement of a vacant manager position on a psychiatric addiction team. The organisational affiliation for that position was decided based on the selected candidate’s profession (nurse or social worker) rather than in advance, when announcing the position. The integrated services were expanded with the opening of a day centre for people with borderline personality disorder.

During 2002 and 2003, the integrated services were further expanded by developing services for elderly people and for some new target groups (e.g. those with long-lasting depression and those who were unemployed or on sick leave due to mental health problems). Furthermore, a joint home-support group with staff from both organisations was proposed. In addition, several structural changes were made in the integrated organisation. For instance, social workers and assistance officers were decentralised and sent to the individual units, and co-location was planned for some administrators. The steering committee proposed further expansion to include a representative for service users who were covered by the law that regulated support and service for people with certain functional impairments.

During 2004 and 2005, actions were taken to further develop the practical work such as by opening a new coach position. The coach would support the managers in designing processes for shared service planning. New groups in need of integrated services were identified: people with Asperger’s disease or ADHD, those at risk of criminality or with addiction problems, asylum seekers with mental health problems, and individuals facing deportation. In addition, the organisation expanded by establishing a neuropsychiatric team and new accommodations for people with double diagnoses. The question was raised over whether to institute a common title – coordinator – for all employees, regardless of profession. This was considered to have a symbolic importance, as it would indicate that cooperation was central and that responsibilities could not be transferred across organisations. The steering committee continued to expand, this time with representatives from child and adolescent psychiatry.

In 2006, efforts were made to overcome evolving macro-level challenges. The integration agreement was revised to further clarify the organisations’ equal status and responsibilities concerning costs. Participation in further education became compulsory for all inter-professional team members, and opportunities for shared research and improvement activities were investigated. The senior county council managers decided to close one of the units. However, to avoid the closure, the municipality took over responsibility for the unit. Another example of integration development was the evolution of the coordinator role into the case manager role, as advocated in the national clinical guidelines on psychosis.

In 2007, a statement was made regarding the importance of giving equal value to the service users’ existential, medical, psychological and social well-being rather than emphasising only some of these depending on the organisation. The steering committee and the stakeholder associations presented a revised vision that highlighted how service users and the two organisations would cooperate to provide flexible and need-based support. This vision also specified the planning, development and evaluation of the units and the teams, emphasising that the professionals’ roles needed to be streamlined so that they would become experts. All service users’ needs were analysed to inform the organisation about whether its services should be revised. Some changes in the needs were noticed; consequently, the units revised their services to better suit people who lacked long histories of inpatient care. The organisation continued to expand by including services for young, self-harming people; the focus was on improving cooperation regarding young people and those with bipolar disorder or complex needs.

In 2008, the integration agreement was once again revised, and the shared routines for risk assessments were included. The steering committee actively requested the stakeholders’ views on the integrated services. They invited partners, stakeholder associations and service-user representatives to discuss these issues. In addition, services continued to be developed for people with neuropsychiatric diagnoses and elderly people with mental illnesses or health problems related to drug abuse and addiction. For the latter, local guidelines clarifying the shared responsibility were developed. The neuropsychiatric staff members were trained accordingly.

In 2009, the stakeholder associations’ roles were strengthened by increasing their participation in the meetings and by gathering their views on the new integration agreement. Substantial work was done to develop integrated services for new user groups. For instance, an initiative was launched to develop collective, overall support for users of the care and habilitation service for people with disabilities. In addition, this service expanded to handle the increasing number of service users with neuropsychiatric disorders. A growing group in need of health and social care comprised traumatised refugees, who required new and well-adjusted integrated services. To treat this group, the organisations co-applied for project funding to develop a care program.

In 2010, continual efforts were made towards the improvement of stakeholder associations. This action was consistent with the search for shared and streamlined service activities focusing on cost reduction. The efforts to increase the service users’ involvement in care and support were also on the agenda. The overall integration agreement was again revised, this time to clarify the steering committee’s responsibilities. In addition, attention was drawn to subgroups that were not yet included in the target population of the integrated organisation. Planning for the housing of people with long-term substance abuse and extensive care needs started with a guiding principle that these individuals would be able to maintain the accommodation regardless of which organisation had the formal responsibility for the individual. Further contacts were made with the primary health care to develop care for elderly people with mental illness, substance abuse and drug addiction. Another central group was people with ADHD, for whom integrated health and social care were considered to be essential. Cooperation was under development with the municipal unit around disabled people and a correctional care unit.

In 2011, the ability to cooperate with primary care was hampered as the number of private care providers increased. Nonetheless, new forms of cooperation within subgroups, such as people with substance abuse and within the neuropsychiatry, were successfully established. In this development work, a mobile team was launched for supporting young people with neuropsychiatric diagnoses, and the care centre for addicts was advanced. The steering committee continued to find solutions to the funding of the integrated services. An example was a new, integrated type of employment form where a manager was formally employed by the municipality, but the costs were shared through the county council purchasing the manager’s services.

In 2012, a regional agreement on how to support people with a mental illness and disability was reached, which strengthened the integration of focusing on the needs and shared responsibilities of service users. The integrated organisation was reviewed in two evaluations. An external evaluation concluded that cost-effective and high quality care was provided and that the steering committee served an important role in overviewing the integration. The internal evaluation underlined that the resources of the relatives and the families could be more optimally used. Consequently, stakeholder associations were invited to take part in discussions of their role and the revision of the services. A new function, a multi-case manager, was also established to handle service users with highly complex needs. Furthermore, clarification of the shared responsibility for service users with psychiatric diagnoses and substance abuse was made, and a newly diagnosed psychiatric patients’ team was initiated to provide integrated services at a care centre for addicts. Further, an operational management group of representatives from the units for adults and elderly people was created and supplemented with a representative from the psychiatry centre.

In 2013, attention was drawn to savings opportunities by further developing the integration. For instance, integration of services with the neighbouring municipalities was discussed. The cooperation agreements were revised regarding elderly users, children and adolescents, and efforts were made to improve cooperation between the psychiatry centre for adults and a centre for children and adolescents to better meet the needs of children, young people and their families. Other areas of improvement included clarifying the primary care and psychiatry responsibilities and reorganising neuropsychiatric care in order to make it more cost-effective. An inter-sectoral co-located neuropsychiatric outpatient clinic was proposed. Despite the economic challenges, the willingness to further integrate health and social care for people with mental illness continued. For example, a “house of health” with all services co-located at one place was planned together with web-based network-gathering activities.

In 2014, the steering committee decided to only meet once per semester. The role of the committee had become more “consultative” and less of a working group due to an increased number of members, which in turn was an effect of the increased number of services included in the organisation. Meanwhile, the steering committee function changed, however, the practical integrated work at the unit levels continued intensively. For instance, procedures for coordinated individual rehabilitation plans were developed, and various improvement projects were launched.

In 2015, attention was again drawn to the needs of elderly service users and to unaccompanied refugee children. Integration with geriatric psychiatry care, primary care and elder care continued to be of high priority but challenging. The economy was strained for both organisations. A new review of the services and the service users’ needs was made. The number of service users in need of health and social care had decreased, while some changes in their needs were also noticed. As a consequence, efforts were made to reduce costs by streamlining the integrated processes. At the end of the year, the steering committee decided to schedule two meetings per semester since the time between the meetings was concluded to be too long.

### Empirical themes

#### Shared structure and ongoing refinement

The services of the municipality and county were interlinked at both structural and functional levels, and the foundation was built on shared mission and agreements, co-leadership and by creating inter-professional teams in co-located services. The question of how the integration would take place was not predetermined; rather, by involving stakeholder associations and other key actors, the organisational development was co-created in a dynamic process through the years. The integration work was characterised by continuous adaptations of interventions on multiple levels. Adjustments were made in order to continuously adapt the organisation and the services to changes in context. For instance, new services were started based on the change in service users’ needs. When financial savings were required, the two organisations streamlined the processes together. All internal improvement work was also made with participation in various improvement projects and with external partners. The thorough work carried out aimed at bringing the municipality and county closer in the pursuit of a shared IT system, aligned steering mechanisms (score cards), service outcome measures, shared routines, common referral forms and shared clinical guidelines. External influences recognised to cause fragmentation were handled by strengthening cooperation, for example, by underlining the equal state of the organisations in the cooperation agreement and by emphasising the equal value to the service users’ existential issues of medical, psychological and social well-being. The following excerpt from the steering committee minutes exemplifies this:

The county council and the municipality are two organizations that complement each other. […] The things we do, we do together. […] The reform requires functioning forms of cooperation and creation of shared goals along with a union of our cultures and decision-making systems. But still, the two cooperating organisations need to continuously develop their own working methods.

### Continuous learning

The integration of health and social care emerged as a fusion of norms, values, assumptions and behaviours from two different sectors, which had certain challenges to overcome. A mutual understanding of the differences, including the mission of each sector, was recognised and respected. Formal structures for learning were created by allowing employees, managers and service users to exchange experiences and knowledge. The financial support of learning activities was strategically used over the years to promote and develop integration. The development of new ways of working such as teamwork and case management was given financial support. Managers’ need for development was also recognised, and their professional development was among other things supported through the coaches who provided support in managing shared service planning. The managers were also innovative in implementing new working methods and functions before these methods and functions were decreed by the national government. Data from all levels of the organisation were continuously collected to assess progress. These measurements enabled immediate reactions, which in turn contributed to continuous optimisation of the conditions for sustainability of the inter-sectoral cooperation. The following excerpt from the steering committee minutes exemplifies this:

The psychiatric reform requires mutual respect and trust for the cooperating health and social care professionals’ different conditions, tasks and methods. Only thus, it is possible to achieve a common set of values upon which the concrete work should rest. In order for this respect and understanding to be maintained, ongoing, mutual knowledge development is required.

### Cooperation as a guiding principle for management

The composition of the steering committee could be described as dynamic since it constantly adapted to match the service users. Permanent members were representatives from the mental health services at the municipality and the county, along with the stakeholder associations. The continuity among the core members was high, and other temporary members were determined by the content of each meeting. The two project managers had a central role for the creation of the integration. They, representing both municipality and county council, made key contributions in the creation of a culture and shared values. Also, the line managers (i.e., co-leaders) functioned as opinion leaders and cultural carriers and therefore had a strong symbolic value. In addition, they worked closely together in applying co-leadership, as all leaders on all levels, and thereby served as role models. The decision-making process was based on dialogue and negotiation, and all solutions were consensus-based. The following excerpt from the steering committee minutes exemplifies this:

Cooperation is necessary to realize the reform. It applies to several levels, county, municipality, team and at the individual level. [...] In our services, service users, municipality and county council cooperate for flexible and needs-based support for the different target groups. The cooperation includes planning, development and evaluation of operations.

### Service user centeredness

Over the years, the steering committee recognised the interdependence of physical, psychological and social factors in health and illness, which was manifested in the committee early on and set the goal to collaborate around each service user. The formal agreements stated that service users should be at the centre. The services were therefore organised around each service users’ personal needs, rather than from the perspective of the organisations. For instance, this was achieved by having ongoing stakeholder involvement through representatives from the stakeholder associations in the steering committee and local service user groups at the service centres. Thus, the service users took part in co-creating the services. On an individual level, coordinators were introduced early on in the process, and a multi-case manager was appointed for service users with complex needs. The content of the care and support was also co-produced according to individual rehabilitation plans, and health and social care interventions were continuously followed up by the use of CAN data. As new service user groups were added over time, new inter-professional teams were arranged to meet the specific needs of the new groups. The following excerpt from the steering committee minutes exemplifies this:

The services should be formed and developed based on the service users’ needs. [...] The organisational and economical conditions should therefore be arranged to enable long-term care and support, based on the service users´ need of continuity.

## Discussion

The study aimed to gain insights into the dynamics of the sustainable change of integrated health and social care. Five main factors were found to be essential for the achievement of the 20 years of inter-sectoral cooperation.

First, the integration was characterised by ongoing adaptations. The services provided and the work of the steering committee was constantly improved based on changes in the surrounding context. The needs of the service users were frequently reviewed in order to create new services or to adapt the existing ones. Service users and stakeholder associations were considered important partners in this. This also applied to the view of other collaborators (e.g., Primary Care, Social Insurance Agency, Public Employment Service etc.) who came to change over time depending on the service users’ needs. The importance of using bottom-up strategies for implementation of new intervention has been emphasised by others [36]. In this case, the steering committee worked actively to align the organisational characteristics with actual needs, meaning that inappropriate structures were removed. This is in line with the DSF, which also recommends contextualising or removing non-outcome-focused intervention components [13].

Secondly, the ambition for ongoing learning among all stakeholders was highly present. Continuous feedback on performance was provided with the measures on the organisational, professional and service user level, which was one step in the creation of a culture for learning. Previous studies on collaboration have shown that arenas for dialogues and exchange of relevant knowledge is important [35], and we found that the decision to co-locate all health and social services in co-run centres required interdisciplinary teamwork on a very practical level, which also surfaced as an important step in the continuous learning and integration of the services. The issue of culture is highly relevant for multi-sectoral collaboration (e.g., between health and social care), since the organisations often have different cultural lenses. In a similar manner, each professional group tends to have its own professional culture, which makes this type of organisation even more multicultural [37]. Protectionism and scepticism towards other professional groups are common in integrated services [38]. The current organisation was no exception to this, but it had a clear ambition on tackling these challenges together through collaboration, education and experience exchange, which has been shown to contribute to successful multidisciplinary integration [39].

Thus, the third issue characterising this sustained integration of services was the emphasis on collaboration. This was one of the most essential guiding principles in the steering group’s work over time. This manifested in considering the dissimilarities, conditions and needs of each organisation in decision making. Thereby, the steering committee was long-term-oriented with collaboration in mind, while also continuously solving the problems at hand to enable collaboration on a practical level. The many actions that the steering committee took initially and the persistent work for the continuation of cooperation reflect a firm belief in inter-sectoral cooperation. These findings relate to prior research showing a relation between committed leaders across the organisation and successful long-term change [40]. The role of managers and leadership, together with the leadership system, is repeatedly highlighted as crucial for change, especially for sustainable change [41]. The leadership system in this case was based on co-leadership and cooperation. The work tasks were regarded as a joint responsibility, which is consistent with previous research on shared leadership showing that a close leadership created space for forward thinking and a long-term approach to work [42].

Fourthly, the service users, their families and the stakeholder associations were key partners in the collaboration and in forming the services. Their engagement in the development of processes was a core strategy used by the steering committee to align interventions to the service users’ needs and to create empowerment. Furthermore, equal importance was given to all of the service users’ needs (medical, psychological, social etc.) rather than prioritising one of these based on sectorial priorities. The shared holistic view on service users and, consequently, an identified need to organise health and social care in a cohesive manner enabled the creation of a shared vision and strategy formulation, which in turn set the direction for the organisation and its priorities. It also seemed that the two organisations could always make up for the needs by putting the service users’ needs first, which helped them to solve potentially sensitive issues in funding and managing the integrated services. This may have caused them to become more solution focused and less protective, enabling the establishment of a shared organisational culture that includes roles, norms and values, which is also underlined by Schein as important [43]. In the research examples of service users being active, the contributors of skills and knowledge in the development of healthcare services can be found [44, 45]. The interaction between service users and health and social care providers forming a partnership is referred to as co-production [46], co-creation [47], experienced based co-design [48] and patient and public involvement [49], to mention a few. Hence, research has shown that the consultative approaches are more common than partnership [50]. However, it seems that this integrated health and social care organisation was an early adopter of this involvement approach enabling service user participation.

Lastly, the steering committee’s work was dynamic, and new members were invited to participate depending on what services were included in the integrated organisation. At the same time, their work was characterised by a low turnover since the core individuals in the steering group were the same during the entire 20-year period. For instance, the minutes were written by the same person in all cases. The stable step of individuals with a shared vision certainly had a great impact on the sustained integration. Furthermore, the two project managers, holding key functions in the process of integration, were the same individuals throughout the years. These functions, called opinion leaders or program leaders in other studies [51], have had a great impact on integration. Previous research has also highlighted the importance of stability on a strategic and operational level to overcome sector-related challenges in integrated health and social care [52]. However, the current study does not reveal whether the impact of the stakeholders derives from what they did, the fact that they were the same people over the years or a combination of these factors.

### Methodological considerations

The main strength of the study was the long time period in which we were able to complete a thorough document analysis. This provided valuable insight into the steering committees’ work over time. A potential limitation with the minutes afforded for study in each meeting was that we were restricted to the information provided in the documents. It’s possible that other data sources such as interviews could have provided a broader picture of the actions taken by the steering committee. Nevertheless, the minutes were rich and provided insights into how the committee members perceived their context, the organisation and their actions rather than only consisting of decisions and actions. As these minutes were not designed with research in mind, the bias from leaving out certain information such as disagreements and conflicts must be considered. Furthermore, the same person wrote out the minutes of the meeting throughout the study period. This can be considered a strength since the minutes followed the same structure and had an equal amount of information throughout the years. At the same time, it can limit the type and information provided since one person made decisions on what to note, although the minutes were always reviewed by the other committee members. Generally, the overall framework helped to identify several factors related to the dynamics of sustainable change. More precisely, the framework was found useful as a tool to limit the scope of relevant data and in interpreting our findings. While the framework focused our attention to some specific components we still strived to remain open for unexpected findings and alternative explanations. One example was the identification of service user centeredness as an enabling factor for the achievement of long-lasting cooperation. By this the services were organised around each service users’ personal needs, rather than from the perspective of the organisations. A limitation to generalisability of the findings can be that the study was conducted in one specific geographical area, which was characterised by long-term integrated health and social care services.

### Suggestions for future research and implications for practice

As this study primarily addresses the steering committee decisions and actions, future research could build on our findings by including perspectives on organisational champions and the meaning of organising networks for achieving sustainability. As the knowledge on champions and networks increases, we stress that organisations and services adjust accordingly. We suggest three main areas of future research: 1) studies on the sustainability of change; 2) studies on the interrelatedness of factors impacting sustainable change; and 3) longitudinal studies on the impact of different factors on sustainability (e.g., to explore the importance of leadership during different stages of change). In regard to practical implications, the findings suggest that service user involvement and the critical review of service users’ needs on a regular basis are essential in order to tailor to the current needs and services. Furthermore the importance of continuously adapting the content of the change to suit its context, was clear, and it’s suggested that continuous refinement of the change content was found to be more important than designing the change at the pre-implementation stage.

## Conclusion

This study provides some valuable insight into the dynamics of sustainable change and the understanding of key managerial actions in order to establish, develop and support the integration of health and social care for people with complex mental health needs. The development of inter-sectoral cooperation was characterised by a participatory approach in which a shared structure was created to support cooperation and ongoing quality improvement and learning focused on the service user’s needs. The key management principle included cooperation on all organisational levels as well as with service users, stakeholder associations and other partner organisations. This study shows that all these parts were interrelated and collectively contributed to the creation of a structure and a culture that supported the development of dynamic and sustainable health and social care.

## Abbreviations

CAN: the Camberwell Assessment of Needs scale
DSF: Dynamic Sustainability Framework
